# System-Based Differential Gene Network Analysis for Characterizing a Sample-Specific Subnetwork

**DOI:** 10.3390/biom10020306

**Published:** 2020-02-14

**Authors:** Yoshihisa Tanaka, Yoshinori Tamada, Marie Ikeguchi, Fumiyoshi Yamashita, Yasushi Okuno

**Affiliations:** 1 Graduate School of Pharmaceutical Sciences, Kyoto University, Kyoto 606-8507, Japan; tanaka.yoshihisa.42n@st.kyoto-u.ac.jp (Y.T.); yamashita.fumiyoshi.3m@kyoto-u.ac.jp (F.Y.); 2 RIKEN Cluster for Science, Technology and Innovation Hub, Medical Sciences Innovation Hub Program, Kanagawa 230-0045, Japan; okuno.yasushi.4c@kyoto-u.ac.jp; 3 Graduate School of Medicine, Kyoto University, Kyoto 606-8507, Japan; ikeguchi.marie.44n@st.kyoto-u.ac.jp

**Keywords:** gene network, differential network analysis, lung cancer survival analysis, EMT

## Abstract

Gene network estimation is a method key to understanding a fundamental cellular system from high throughput omics data. However, the existing gene network analysis relies on having a sufficient number of samples and is required to handle a huge number of nodes and estimated edges, which remain difficult to interpret, especially in discovering the clinically relevant portions of the network. Here, we propose a novel method to extract a biomedically significant subnetwork using a Bayesian network, a type of unsupervised machine learning method that can be used as an explainable and interpretable artificial intelligence algorithm. Our method quantifies sample specific networks using our proposed *Edge Contribution value* (ECv) based on the estimated system, which realizes condition-specific subnetwork extraction using a limited number of samples. We applied this method to the Epithelial-Mesenchymal Transition (EMT) data set that is related to the process of metastasis and thus prognosis in cancer biology. We established our method-driven EMT network representing putative gene interactions. Furthermore, we found that the sample-specific ECv patterns of this EMT network can characterize the survival of lung cancer patients. These results show that our method unveils the explainable network differences in biological and clinical features through artificial intelligence technology.

## 1. Introduction

The use of high throughput technologies in molecular biology has led to the generation of large volumes of data, and thus, the development of precise methods for handling such large data is required. Understanding the cellular mechanisms at a system level forms a fundamental goal of these methods. Elucidation of the cellular mechanisms is indispensable for discovering biologically significant events, especially for clinical applications like finding new drug targets [1].

Researchers have developed several approaches to elucidate cellular mechanisms. One of the most popular methods is the conventional mRNA expression analysis to identify Differentially Expressed Genes (DEG) [2,3]. This method extracts independent genes using differences of expression levels between different conditions; e.g., control and perturbated samples. Likewise, pathway analysis maps genes onto known pathways to classify genes based on their enrichment in the maps of well-known pathways, such as the EGFR signaling and DNA damage repair pathways [4,5,6]. These traditional approaches, however, are unable to discover *de novo* pathways and relationships.

Network analysis constitutes a promising method to interpret the big omics data, and researchers have applied it to many biomolecular investigations [7,8]. However, since the gene network estimation brings in a huge number of putative gene regulatory relationships often expressed as a hairball [9], establishing a method for identifying biologically significant subnetworks or inter-gene relationships from such a large and complicated network has been challenging. Established methods that identify regulatory relationships in large gene networks mainly adopt two approaches: one focuses on hub genes that have a large number of connections [10,11,12,13], whereas the other, known as differential network analysis [14], compares network structures derived from different conditions. While the former approach fails to extract edges regardless of the gene network analysis, the latter requires individual networks reflecting different conditions. Nonetheless, it remains difficult to satisfy sufficient sampling to generate data-driven and condition-dependent networks. For instance, our study uses a data set that consists of only three samples per condition, which makes a Bayesian Network (BN) and structure-based approach unfeasible.

**Figure 1:**
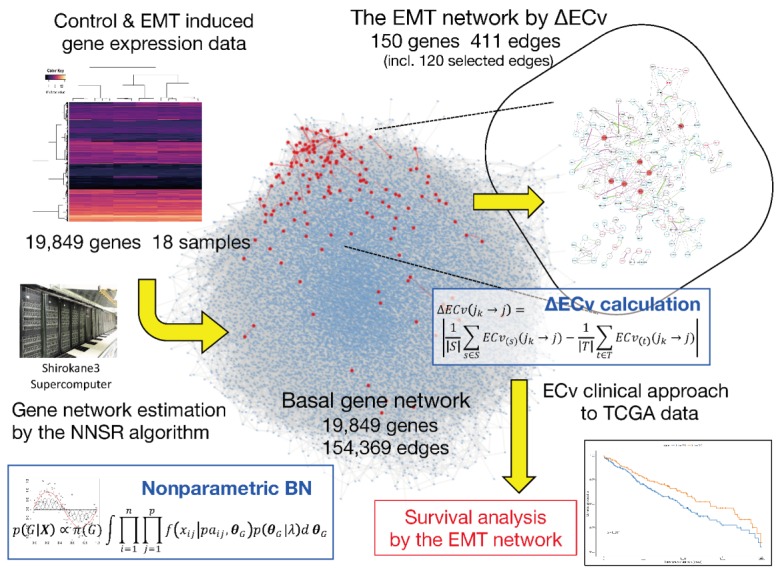
Overview of our proposed method. The center hairball (blue) is the basal network. The red nodes in the basal network represent the ΔEdge Contribution value (ECv)-extracted Epithelial-Mesenchymal Transition (EMT) network.

Herein, we propose a novel method to extract a biomedically differentiated subnetwork from a huge number of edges in a gene network estimated using a relatively small number of samples. Compared to the structure-based network analysis, our approach aims to extract edges, which represent the system-level differences between the samples in cellular networks without comparing network structures. Therefore, even with a limited number of samples for conditions of interest, our method will extract system-based differential networks. The outline of the proposed method is shown in Figure 1. Following the reported method [15], a gene network from the available gene expression data sets was estimated using nonparametric BN. This generates a basal network with more than a hundred thousand edges between approximately 20,000 genes. Most importantly, our method calculates an *Edge Contribution value* (ECv) for every single edge in a gene network with respect to each sample. The proposed ECv can quantify a particular edge for each sample using the estimated model. Therefore, based on the differences in ECvs between different samples, which imitate biologically or clinically-specific phenomena, our method highlights the differentiated subnetwork. This method defines the edges out of a possible hundred thousand links between genes, and thus demonstrates major differences in regulating the basal gene network potentially associated with target diseases or phenomena. Since the extracted subnetwork highlights specific differences between samples, it may portray expression data sets that are not used in network estimation or subnetwork extraction. We proved that the network ECv pattern with respect to a patient sample for certain types of diseases can be adopted for clinical classifications by validating real data.

The idea of estimating sample specific gene networks has already been addressed. For instance, Shimamura et al. (2011) [16] proposed a structural equation-based model that estimated sample specific regulator-modulator-target relationships. Their method assumes the gradual effect of modulators to parent-child relationships throughout the collected samples. Thus, this method generally requires sufficient samples to detect such relationships. Yu et al. (2015) [17] tried to extract personalized gene networks based on their differential network model. Their model assumes an existing network, such as the protein-protein interaction network, and combines it with genes extracted by traditional methods such as DEG and gene pairs that are independently evaluated by their novel differential expression covariance method on that network. Kuijjer et al. (2019) [18] proposed a method to estimate sample-specific regulatory networks where the linear combination of edge weights in particular networks forms the aggregated network. Unlike these existing methods, our proposed method estimates a globally optimized structure as a cellular model with the BN model and correlates edge differences to the samples using the estimated model parameters. None of the existing methods realize extraction of the edge from a limited number of samples and a large network except for our method.

We examined our method on Epithelial-Mesenchymal Transition (EMT) [19] to understand its process through the representative EMT subnetwork; we then applied it to The Cancer Genome Atlas project (TCGA) clinical data [20]. In this analysis, the basal network was estimated from the small number of samples mimicking EMT in lung cancer cell lines. By comparing ECvs between EMT-induced and control samples, we succeeded in extracting the EMT-characterized network. In addition, we applied this EMT network to TCGA clinical data to test if the network can generate a prognosis profile, as EMT is a major factor involved in the prognosis of cancer patients. Our results show that the prognosis for lung cancer patients was partially associated with the ECv patterns in the EMT network, according to the survival analysis. This indicates that our proposed method correctly extracts a subnetwork determining patients’ EMT characteristics from a large number of nodes and edges in the network.

## 2. Materials and Methods

### 2.1. Nonparametric Bayesian Network

As described in the Introduction, we used nonparametric BN to estimate a gene-to-gene regulatory system from gene expression data [21]. The BN estimation is an unsupervised machine learning algorithm that is able to capture cause-and-effect relationships among variables from its observations by optimizing the global structure of the network. Therefore, it constitutes an ideal method to estimate gene regulatory systems and has been successfully applied to many gene network analyses [12,22,23]. In this section, we provide a brief explanation of the model.

Let ***X*** be an *n*-by-*p* data matrix whose element $x_{ij}$ corresponds to the observation of the *j*-th variable; that is, the expression value of the *j*-th gene, at the *i*-th sample, where *n* represents the total number of samples and *p* the number of variables. In the nonparametric BN model, we consider the joint density of all the variables and assume that it can be decomposed as the product of the local conditional densities, such as

(1) $$f\left(x_{i1},\ldots ,x_{ip};\theta_{G}\right)=\prod_{j=1}^{p}f\left(x_{ij}|{pa}_{ij}^{G}\left(x_{ij}\right);\theta_{j}\right),$$

where ${pa}_{ij}^{G}\left(x_{ij}\right)=\left(pa_{i1}^{\left(j\right)},\ldots ,pa_{iq_{j}}^{\left(j\right)}\right)$ is the set of observations in the *i*-th sample of $q_{j}$ dependent variables of the *j*-th variable, and $\theta_{G}=\left(\theta_{1},\ldots ,\theta_{p}\right)$ are the parameters of the conditional densities. This decomposition can be represented by a directed acyclic graph consisting of nodes representing variables and edges connecting the nodes called “children,” to their dependent variables called “parents.” To model parent-child relationships, we employ *B*-spline nonparametric regression. In the model, the gene expression is represented by

(2) $$x_{ij}=m_{1}^{\left(j\right)}\left(pa_{i1}^{\left(j\right)}\right)+\ldots +m_{q_{j}}^{\left(j\right)}\left(pa_{iq_{j}}^{\left(j\right)}\right)+\varepsilon_{j},$$

where $m_{k}^{\left(j\right)}\left(pa_{ik}\right)=\sum_{l=1}^{M}\gamma_{lk}^{\left(j\right)}b_{lk}^{\left(j\right)}\left(pa_{ik}^{\left(j\right)}\right)$ for $1\leq k\leq q_{j}$ and $\varepsilon_{j}∼N\left(0,\sigma_{j}\right)$. Here, $b_{lk}^{\left(j\right)}\left(\cdot \right)$ is a third-order *B*-spline function which is determined by the range of the observations and $\gamma_{lk}^{\left(j\right)}$ is its coefficient. *M* is the number of the *B*-splines and we use M=20 as used in Imoto et al. (2002) [21]. The local density in Equation (1) can be written as

$$f\left(x_{ij}|{pa}_{ij}^{G}\left(x_{ij}\right);\theta_{j}\right)=\frac{1}{\sqrt{2\pi \sigma_{j}^{2}}}\exp\left[-\frac{{\left\{x_{ij}-\sum_{k=1}^{q_{j}}\sum_{l=1}^{M}\gamma_{lk}^{\left(j\right)}b_{lk}^{\left(j\right)}\left(pa_{ik}^{\left(j\right)}\right)\right\}}^{2}}{2\sigma_{j}^{2}}\right],$$

where $\theta_{j}=\left(\gamma_{1,1}^{\left(j\right)},\ldots ,\gamma_{M,q_{j}}^{\left(j\right)},\sigma_{j}^{2}\right)$ is the parameter vector for the local density to be estimated from the observations. The network structure can be determined based on the maximization of the marginal posterior

$$p\left(G|X\right)\propto \pi \left(G\right)\int \prod_{i=1}^{n}f\left(x_{i1},\ldots ,x_{ip};\theta_{G}\right)\pi \left(\theta_{G}|\lambda \right)d\theta_{G},$$

where *G* represents the network structure, π(G) the prior probability of *G* and $\pi (\theta_{G}|\lambda )$ the prior distribution of $\theta_{G}$. Here λ is the hyperparameter vector determined also by the maximization of the posterior. Searching for the optimal structure of the BN is known to be an NP-hard problem. Thus, we use the Neighbor Node Sampling and Repeat (NNSR) algorithm [15] that is applicable to more than twenty thousand genes for obtaining an approximated structure of the huge BN. We call this network *the basal network* in the later steps.

### 2.2. Proposed Method for Evaluating Sample Specific Edge Contribution Values

Our basic idea is to evaluate a single edge value of an observed sample through the mathematical model estimated as a BN. In the model, the expression value of a child gene is represented by a linear combination of their parent values transformed by the functions denoted as $m_{k}^{\left(j\right)}\left(\cdot \right)$ in Equation (2). Considering this nonparametric regression function, $m_{k}^{\left(j\right)}\left(pa_{ik}^{\left(j\right)}\right)$ can be regarded as a contribution of the *k*-th parent of the *j*-th gene to the expression value of $x_{ij}$, because these values of $q_{j}$ parents constitute the value of their child. According to this, we define *Edge Contribution value* (ECv) of edge $j_{k}\to j$ with respect to the *i*-th sample as

$$ECv_{\left(i\right)}\left(j_{k}\to j\right)=m_{k}^{\left(j\right)}\left(pa_{ik}^{\left(j\right)}\right),$$

where $j_{k}$ represents the index of the *k*-th parent of the *j*-th gene.

**Figure 2:**
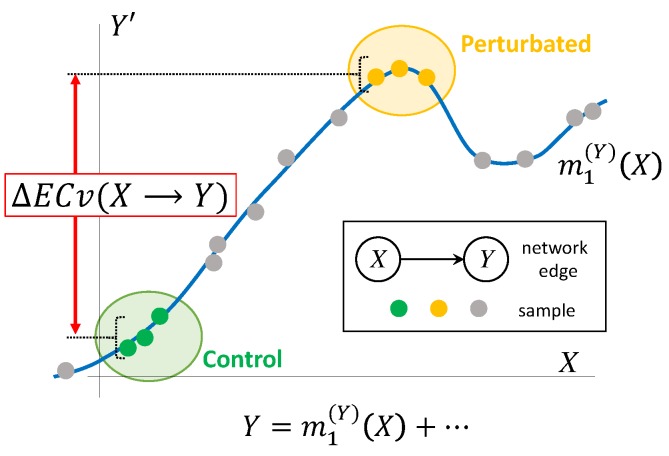
Graphical representation of ΔECv. Line (blue) is a nonparametric regression curve $m_{1}^{\left(Y\right)}\left(X\right)$ for edge X→Y estimated with Bayesian Network. Plots for *X* axis correspond to actual mRNA signal values and $Y^{\prime }$ axis partial residuals where the effects of the other parents are subtracted from the children’s mRNA signal values. Plots (green) and plots (yellow) represent, for instance, control and perturbated samples, respectively. Plots (grey) represent other values used for determining the regression curve. Values in $Y^{\prime }$ axis also stand for output through the regression function with parents’ mRNA signals. By the definition, these correspond to ECvs. ΔECv is defined as the difference between two conditions. In this example, the difference of ECvs between perturbated and control samples, i.e., the ΔECv of these two conditions, is depicted.

Since ECv is calculated from the model parameters to every single edge in the estimated network, the ECv of an edge can be considered as its edge weight, representing how it contributes to the link between a node pair for a certain sample. However, this calculation of ECv alone does not represent an evaluation of the absolute importance of the edge, because it is impossible to determine the size of calculated ECv. This is similar to gene expression values where differential expression is considered. Therefore, two ECvs of an edge must be compared between samples. For instance, let us assume that there are control and drug-perturbated samples in an in vitro cell assay. To obtain the differential edge in terms of ECv, we define a variation of ECv, ΔECv, as

$$\Delta ECv\left(j_{k}\to j\right)=\left|\frac{1}{\left|S\right|}\sum_{s\in S}ECv_{\left(s\right)}\left(j_{k}\to j\right)-\frac{1}{\left|T\right|}\sum_{t\in T}ECv_{\left(t\right)}\left(j_{k}\to j\right)\right|,$$

where *S* and *T* are sets of indices of samples observed in particular conditions, respectively. The graphical representation of ΔECv is shown in Figure 2.

The ΔECv of an edge is the absolute difference that stands for the difference in edge contribution between samples of different conditions. Thus, an edge that shows significant ECv difference between certain samples represents a distinctive edge. In our experiment, ΔECv implementation did not result in a large number of candidate edges that were needed for following the investigation. This suggests that ΔECv functions as a screening method for candidate networks from the estimated basal networks that are important under certain conditions. The network estimation with a small number of samples generally increases the number of false positive edges due to spurious correlations between variables. It is expected that the ΔECv can overcome this problem, because ΔECv might highlight a sample difference which is determined by not only the estimated model but also the sample-specific differences through the estimated model. Note that the number of samples in *S* and *T* here can be 1 because we focus only on differences of ECv so that only a single pair of samples is required to extract edges.

As a consequence of ECv calculation, we obtain a matrix consisting of ECvs for all of the edges and samples. We call this *ECv matrix*. More precisely, the ECv matrix ***E*** is an *n*-by-*m* matrix whose element $e_{iv}$ corresponds to ECv of the *v*-th edge at the *i*-th sample, where *m* represents the total number of estimated edges and *n* the number of samples. The ECv matrix thus can be considered as a set of each sample’s ECvs. Since ECv originally represents the sample-specific profiling for each estimated edge, clustering of the ECv matrix highlights differences according to ECvs for each sample, which then allows for the grouping of samples based on their system-level similarities.

### 2.3. Data Preparation

The microarray data for EMT analysis were acquired from Gene Expression Omnibus (GSE49644) [19]. The data set is composed of three human Non-Small Cell Lung Cancer (NSCLC) cell lines: A549, HCC827 and NCI-H358. The microarray experiments were replicated 3 times for both control and TGF*β*-treated cells. Thus, the data consists of 3×3×2=18 samples in total and 19,849 genes. The log_2_-transformed values of preprocessed data were applied to BN estimation and ECv calculation. The clinical and RNA-seq data of lung cancer patients [20] were acquired from the Genomic Data Commons Data Portal at TCGA and UCSC Xena [24]. NSCLC patients with either Lung Squamous Cell Carcinoma (LUSC) or Lung Adenocarcinoma (LUAD) were selected. The patients were first screened to obtain tumor specimens. RNA-seq data was filtered to remove genes with a mean percentile lower than 15, resulting in 17,450 genes. In the clinical data, we removed entries for patients whose follow-up or decease data was more than 2000 days. Further to these preprocesses, we deleted the patient data that were not common in the RNA-seq and clinical data. The number of the final patients for analyses was 426 (alive: 238, deceased: 188) for LUSC and 457 (alive: 285, deceased: 172) for LUAD. The details of the acquired data above are listed in Table S2.

### 2.4. Differential Expression Gene Analysis

The differential expression gene analysis for GSE49644 microarray data was performed using R package limma [25]. Benjamini-Hochberg method was applied for calculation of False Discovery Rate [26].

### 2.5. Molecular Function Analysis

The functional analysis was generated through the use of Ingenuity Pathway Analysis [27].

### 2.6. ECv Matrix Clustering and Survival Analysis

Unsupervised-clustering for the ECv matrix was performed using the “ward.D2” clustering method with Euclidean distance for both edges and samples in R. The following survival analysis was performed with log-rank test using R package surveminer and TCGAbiolinks [28,29].

### 2.7. Network Analysis and Visualization

The network visualization and a part of network analysis were performed using Cytoscape [30].

### 2.8. Computation Environment

All the computation for the network estimation and the ECv calculations in this study were performed by the SHIROKANE supercomputer system (Shirokane3) at Human Genome Center, the Institute of Medical Science, the University of Tokyo, where the computation nodes were equipped with dual Intel Xeon E5-2670 v3 2.3GHz CPUs and 128GB memory per node.

## 3. Results

### 3.1. Basal Gene Network Estimation

For the basal gene network estimation using the BN, we adopted the NNSR algorithm [15] as described in Materials and Methods. The algorithm repeatedly iterates subnetwork estimations in parallel many times for gene sets extracted by the neighbor node sampling method, and determines a final network structure by incorporating edges whose estimated frequencies are greater than the cutoff threshold. The frequencies correspond to strengths of edges in terms of stability or confidence of them. To begin with, we tuned these parameter settings for our analysis. As for the cutoff threshold, since the number of extracted edges was fixed with another quantitative measurement after the network estimation, we employed a threshold of 0.1, which is slightly relaxed comparing the default setting (threshold of 0.2), to include weak putative edges in this step. Although the algorithm can estimate networks for more than ten thousand genes, it supposedly requires more than a hundred samples for an input data. Therefore, we needed to confirm if the algorithm would work with an extremely small number of samples; i.e., 18 samples in our case. For this purpose, we defined the degree of concordance between two networks as the ratio of edges that are estimated in both networks to the total number of edges, and then we tested whether the algorithm produced stable results using this degree. Following the recommendations of Tamada et al. (2011) [15], we performed the network estimation three times with 10,000 times as the number of iterations (we denote this as “T”) of the subnetwork estimation, and calculated the averaged degree of concordance for every estimated-network pair. As a result, the degree of concordance was 72.7%, suggesting that the algorithm could not produce stable gene network structures (Table 1). Therefore, we assessed whether the increased T resulted in stable network structures with the EMT data set. We performed identical evaluations as above for T = 100,000, 500,000 and 1,000,000. We found that T = 1,000,000 was sufficient for our network analysis owing to reduced error rate below 5% and the stable networks with just 18 samples. The results are summarized in Table 1. The final network structure consisted of 154,369 edges with a threshold of 0.1 (Table S1) and the node degree average of 15.55. This basal network is shown in Figure 1. When we used our data set consisting of 19,849 genes and 18 samples, the computation time required for this network estimation was 7 h 55 m 42 s at T = 1,000,000 using 64 CPU cores.

### 3.2. ΔECv Highlights the EMT-Characterized Edges

**Figure 3:**
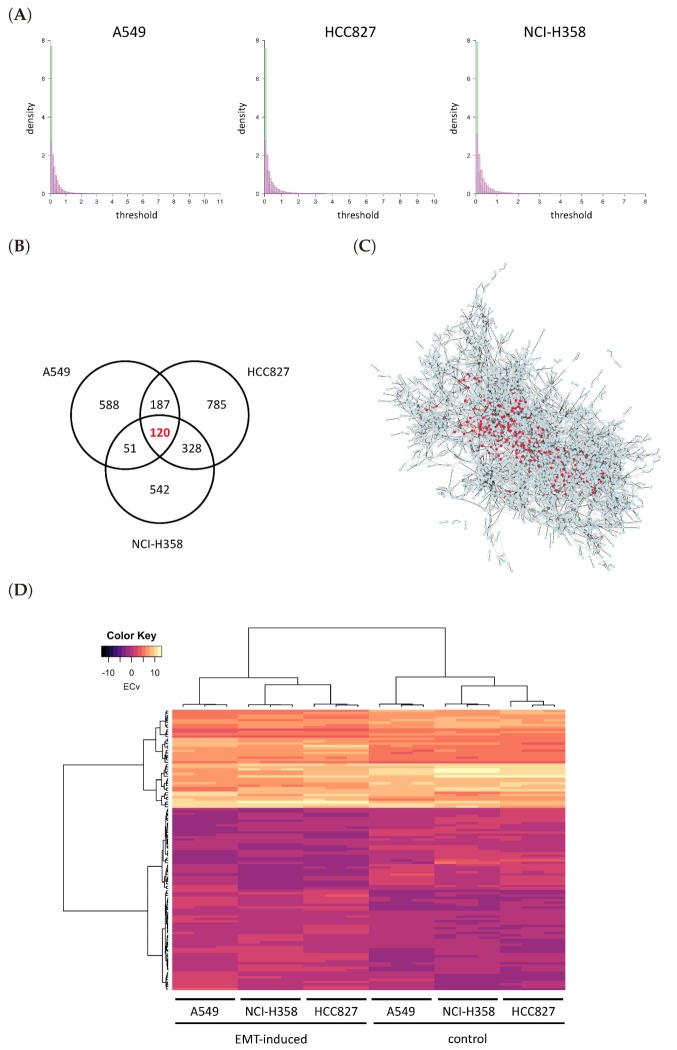
ΔECv analysis. (**A**) The histograms of absolute ΔECv (green) and absolute log_2_ Fold Change (FC) (magenta) for each cell line. FC is defined as TGF*β*-treated/control. The number of total edges is 154,369 for ΔECv. The total number of genes for log_2_FC is 19,849. *Y* axis stands for density. *X* axis corresponds to the threshold for each ΔECv and log_2_FC. (**B**) The Venn diagram represents the numbers of ΔECv-extracted edges for all the cell lines with threshold 1.0. (**C**) The subnetwork of all the 2601 edges which were extracted from each cell lines by ΔECv. The nodes (150) and edges (120) with red highlight the common edges for each cell line. (**D**) Heat map and the result of hierarchical clustering for the ECv matrix of ΔECv-extracted 120 edges with 18 samples.

To examine if our proposed method is applicable to real data, we applied an ECv calculation to the basal network. The microarray data cells were treated with TGF*β* to induce EMT. EMT is a cellular process in which metastasis and invasion are involved [31]. Although EMT has been well-investigated and many EMT-related genes were identified, its molecular mechanism is not fully understood. Once cancer cells invade tissues, this is critical for treatment of cancer and even more so for patient prognosis. Therefore, we aimed to extract an EMT-characterized subnetwork which represents a putative core mechanism of EMT as a cellular system modeled by the nonparametric regression. Compared to control cells, TGF*β*-treated cells represented the EMT profile, which was confirmed by the alteration in cellular morphology and expression levels of EMT markers in the previous study [19]. Following ECv calculation, a 18-by-154,369 ECv matrix was obtained. Since the samples were replicated three times for both control and EMT-induced conditions over three cell lines, we calculated ΔECv with respect to each cell line as

$$\Delta ECv^{\left(u\right)}\left(j_{k}\to j\right)=\left|\frac{1}{3}\sum_{s\in \{\mathrm{EMT}-\mathrm{induced}\}}ECv_{\left(s\right)}^{\left(u\right)}\left(j_{k}\to j\right)-\frac{1}{3}\sum_{t\in \{\mathrm{control}\}}ECv_{\left(t\right)}^{\left(u\right)}\left(j_{k}\to j\right)\right|,$$

where *u* = A549, HCC827 and NCI-H358. The edges that were assigned high ΔECv can be considered as significant differences in the cellular system between control and EMT-induced samples in each cell line. The distribution of ΔECv values for each cell line is displayed in Figure 3A. As shown, most edges do not show significant differences between the two conditions. The number of edges with ΔECv more than 1.0 for A549, HCC827 and NCI-H358 were only 946, 1420 and 1041, respectively, out of 154,369. Given that the total number of edges in the estimated network is 154,369, ΔECv filters out edges which are assumed to be significantly different in EMT. To compare the log_2_ Fold Change (FC) distribution of mRNAs, which is a standard indicator in DEGs, we overlapped their histograms (Figure 3A). This showed that the distribution of ΔECv is much steeper than that of log_2_FC throughout the thresholds, suggesting that ΔECv can be considered as a better indicator for extracting condition-dependent edges. We set ΔECv threshold as 1.0 because it corresponds to approximately top 0.1% of edges out of the total number of edges. To gain reliable EMT-distinctive edges, we extracted 120 edges which exceeded the threshold in all of the three cell lines that are composed of 150 genes (Figure 3B,C). Because there were nine samples both for TGF*β*-treated and control cells, we can evaluate statistical significance of the extracted edges. We performed *t*-tests for ECv between TGF*β*-treated and control samples, and found that 108 edges out of ΔECv-extracted 120 satisfied the criteria of FDR (False Discovery Rate)-corrected *p* value < 0.01 (Figure S1). This supports that ΔECv extracted edges are statistically significant, even though we estimated the basal network from a small number of samples. Furthermore, considering that we used log_2_-transformed expression data, threshold 1.0 for ΔECv was generally supposed to be a 2-FC cutoff for the estimated system. Therefore, we hypothesized that 1.0 was a moderate threshold for the EMT data set and used this in the following analyses. The ΔECv heat map reflects the samples’ distinctive ECv matrix for the selected 120 edges (Figure 3D). This shows that the EMT-induced and control samples were clearly separated into two clusters, which further suggests that our ECv method captures the EMT-induced *pattern* of cellular network differences.

### 3.3. ΔECv Unveils the EMT Networks

**Figure 4:**
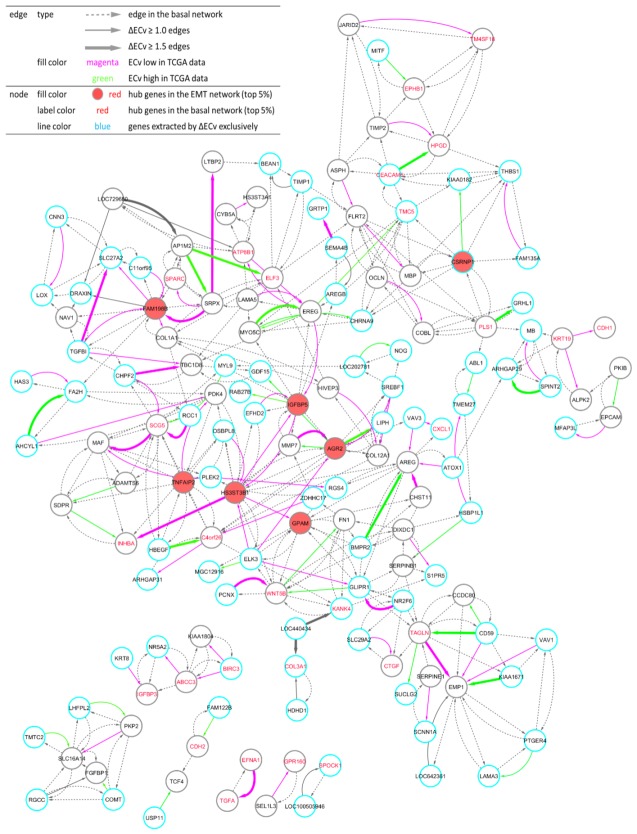
Visualization of the EMT network. One hundred and fifty nodes and 411 edges constitute the network. The total number of connected components is seven. Node: The top 5% hub genes (filled with red) in the EMT network, and the top 5% (labeled with red) hub genes in the basal network are displayed. Nodes (blue line) represent genes extracted by ΔECv exclusively. Edge: Bold edges (grey) and standard edges (grey) represent ones with absolute ΔECv more than 1.5 and 1.0, respectively. ECv high 38 (green) and low (magenta) 70 edges in TCGA data-fitting experiment (Figure 6A and Figure S3B) are labeled. Dotted edges (grey) originally belonged to the basal network.

Using these ΔECv-extracted edges, we aimed to build and visualize a network. We mapped 120 edges and linked the edges that are absent in ΔECv using their connections in the basal network, resulting in the establishment of the EMT-characterized subnetwork (hereafter referred as *the EMT network*) (Figure 4). Eventually, this network comprised 150 nodes and 411 edges. Remarkably, the EMT network shared many nodes of ΔECv-extracted edges; i.e., these edges were linked to each other. The biggest connected component in the 120 ΔECv-extracted edges consisted of 54 edges with 50 nodes. Many other connected components or independent edges were also connected to the biggest one within a single edge of the basal network, whereas only few edges were completely isolated. The inclusion of the basal network edges resulted in the connected component consisting of 371 edges with 127 nodes in the EMT network. Additionally, if we highlighted the ΔECv-extracted nodes and edges in the basal network whose node layout was arranged only using its topological structure, we observed that those were closely located and seemed to constitute a module in the basal network (Figure 1). Furthermore, we mapped top 5% rank hub genes for the basal network and the EMT network to compare their topological localizations in the EMT network and identified 28 of the 1156 genes as the ECv-extracted genes (Figure 4). Interestingly, these basal hub genes did not function as hub genes in the EMT network.

### 3.4. Biological Validation for the EMT Network with the Comparison between ΔECv and DEG

**Figure 5:**
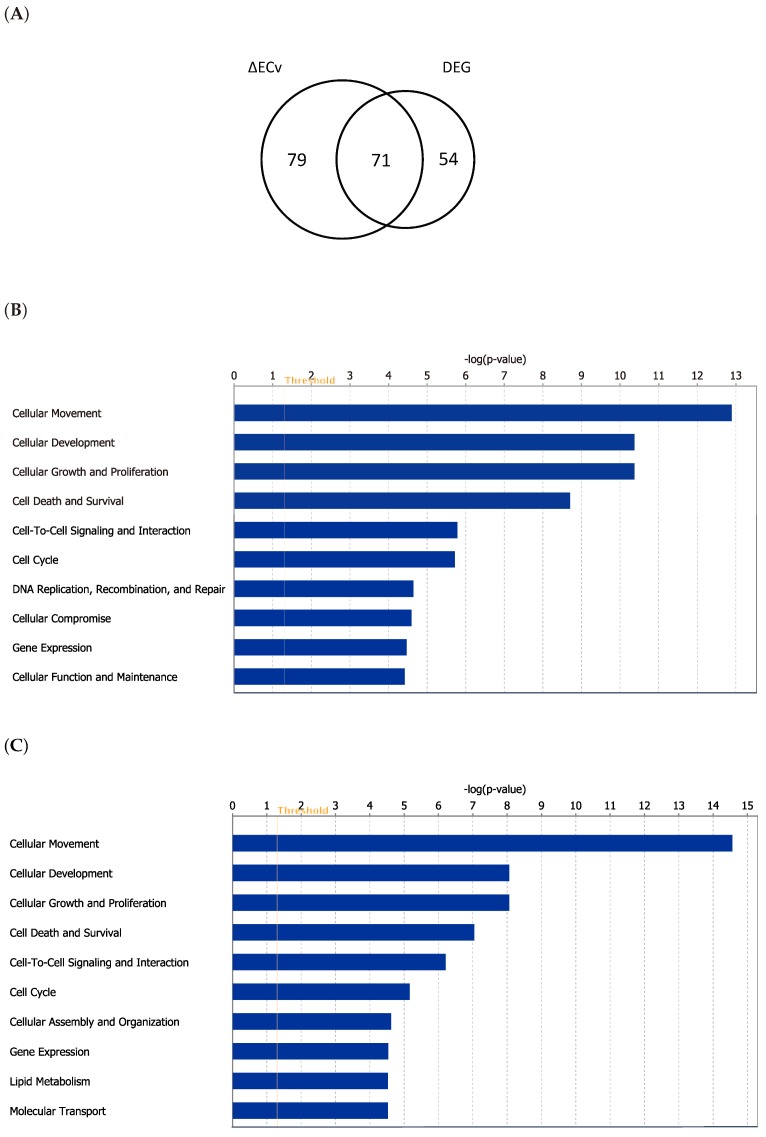
Comparison of ΔECv and Differentially Expressed Genes (DEG). (**A**) Venn diagram for the genes extracted by ΔECv and DEG. (**B**) Top 10 terms of the molecular function analysis for the ΔECv-extracted 150 genes. (**C**) Top 10 terms of the molecular function analysis for the 125 genes through DEG.

To investigate the extent to which ΔECv-extracted genes explain the EMT features biologically, we conducted a method-based comparison between ΔECv and DEG. The DEG analysis was performed by a criteria of absolute log_2_FC > 2 and FDR-corrected *p* value < 0.00001, approximately following a previous report [19], resulting in 125 genes (Figure S2). This DEG-extracted gene set principally reflects a difference between control and TGF*β*-treated samples. The number of shared genes obtained by ΔECv and DEG was 71 (Figure 5A), suggesting that some population of the ΔECv genes belongs to the DEG-extracted gene set. Considering that 2-FC is a standard lowest cutoff for making a decision for potential DEGs, the remaining 79 genes for ΔECv and 54 genes for DEG might be exclusive for each method (Figure 5A). This implies that network-driven ΔECv can extract genes that the conventional DEG method never does. To get more of an insight into the biology involved, we examined whether biological functions are different between the gene sets obtained by these two methods. The molecular functions in the top 6 ranks out of 10 are exactly the same between them (Figure 5B,C), and the top 3 functions of “cellular movement”, “cellular development” and “cellular growth and proliferation” might represent the EMT features. This further supports that at least the major population of ΔECv-extracted genes consists of EMT-related genes. Moreover, we observed that representative EMT markers, *CDH1* and *CDH2*, were included in the EMT network (Figure 4). These results show that our method enables us to identify genes that are not identified through DEG, along with a biological validity, indicating the advantage of our ΔECv method.

### 3.5. A Clinical Approach Using the EMT Network

**Figure 6:**
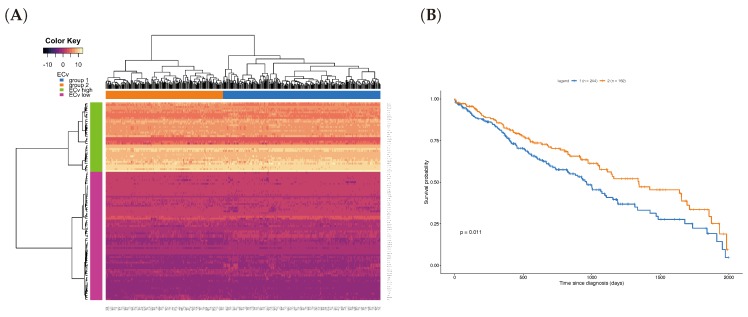
Unsupervised clustering and survival analysis for Lung Squamous Cell Carcinoma (LUSC). (**A**) Heat map with hierarchical clustering for the ECv matrix of 108 edges with 426 samples in LUSC RNA-Seq data. (**B**) Kaplan-Meier curves for the two patient groups; group 1 (blue, n: 244) and group 2 (orange, n: 182), corresponding the patient clusters in the heat map in A. The survival analysis was performed using log-rank test for *p* value calculation.

Finally, considering that ΔECv enables us to emphasize network differences between normal and clinically relevant samples, we attempted to investigate whether the pattern of ΔECv-extracted edges over patients’ samples identifies their properties regarding their EMT network suitability. We directly applied the EMT network on the RNA-seq data of the two types of lung cancer (LUSC and LUAD) patients by calculating their own RNA-seq specific ECv as

$$ECv_{\left(i\right)}^{\prime }\left(j_{k}\to j\right)=m_{k}^{\left(j\right)}\left(pa_{ik}^{\prime (j)}\right)$$

where $pa_{ik}^{\prime (j)}$ represents the RNA-seq expression of the *k*-th parent of the *j*-th node in the basal network, and *i* the patient index in the TCGA data set. Note that $m_{k}^{\left(j\right)}\left(\cdot \right)$ here is the same as the one estimated for the 18-sample microarray data, and we calculated them for only the ΔECv-extracted edges. This experiment tried to reassign the EMT network, which was obtained by the microarray data, to a new RNA-seq data. We hypothesized that it can also highlight the EMT network in the RNA-seq data. Due to the differences in the total number of genes between the microarray and RNA-seq data, we performed this on the 136 genes shared with both EMT network and RNA-seq to gain an RNA-seq-derived ECv, resulting in 108 edges out of 120 edges. Therefore, this ECv calculation produces the 426-by-108 and 457-by-108 ECv matrix for both LUSC and LUAD. The ECv patterns of these matrices were shown as a heat map along with the result of the unsupervised clustering analysis (Figure 6A and Figure S3A). The 108 edges were classified into ECv high and low clusters, indicating that these are involved in network gene up or down-regulation. Since the patients were clearly clustered into two groups, we considered that these groups highlight a system difference in the process of EMT. Given that the metastasis and invasion that is a consequence of EMT is fatal for patients’ cancer prognosis, in order to ask whether these two groups are clinically different, we performed a survival analysis. The result showed that, although this failed (log-rank test *p* = 0.1) for LUAD (Figure S3B), these two groups were significantly different (*p* = 0.011 < 0.05) in terms of their prognosis for LUSC (Figure 6B), suggesting that the LUSC patients were distinguished into groups of better and worse prognosis by their ECv patterns based on their EMT network differences.

## 4. Discussion

Here, we report a novel method to obtain a sample-specific subnetwork using ECv derived from the estimated parameters of the BN. We investigated the application of this method in EMT biology and clinical data analysis. Our method shows the potential ability of capturing the subnetwork and the patient-specific ECv patterns, and these patterns further characterized the prognosis. The prognosis in cancer patients depends on more than the metastasis. This is probably one reason why the combination of clustering and survival analysis works on LUSC, but not LUAD. On the other hand, the results of conventional clustering analysis on ECv matrices showed clear discriminations into two groups both on cell line microarray data and patient tumor sample RNA-seq data. Therefore, our results imply that individuals are distinguished through network differentiation using the proposed quantification of the edges.

Although translating laboratory experiments into the clinical context remains a big hurdle, this study indicates that our approach could be a powerful tool for bridging the divide between them. However, if we use it for this purpose, in vitro experimental design should at least relate directly to practical clinical realities. In addition, because some data sets in replicate experiments involving in vitro assays are markedly homogeneous, we calculate ΔECv using mean ECv difference for each condition-specific sample set in this study. This, however, would not be appropriate for a heterogeneous data set because mean ECv is not supposed to reflect a representative ECv for a particular set of samples. These issues are current limitations of our proposed method and can be improved in the future work.

As discussed previously, existing gene network analyses generally focus on hub genes that are supposed to catalog important regulatory genes in a network. In the EMT network, most of the basal network hub genes were either located at the corners of the EMT network or completely isolated from the biggest connected component (Figure 4). Only a few of these hubs are at the network’s center, suggesting that the basal network hub genes are the master regulators responsible for various cellular processes in the basal network, but not in specific functional modules in the EMT network, as discussed below. This may imply that the major difficulty in hub gene analysis lies in the acquisition of significant genes, especially for samples relating to specific conditions. However, this may also suggest that the network estimation involves appropriate consideration of all the system-level cellular features, even when the number of samples are very small and are measured for a specific condition.

Within the biggest connected components in Figure 4, *HS3ST3B1*, *FAM198B* and *IGFBP5* are the three top hub genes of the EMT network. Genes farthest from the hub genes were closely linked, suggesting that the subnetwork centered on the hub genes essentially represents EMT profiles depicted in the EMT data set. In particular, we found that these components are more enriched in Extracellular Matrix (ECM) genes. ECM constructs a multilayer scaffold structure located outside of the cell membrane and functions as an attachment between cells and tissues, assisting in cell growth, movement, development and differentiation [32]. Given that collagen, proteoglycan and Glycosaminoglycans (GAGs) constitute ECM, it is reasonable that ECM-functional genes, *HAS3*, *FN1* and *MMP7* are located proximally to the top three hub genes. *HS3ST3B1* encodes an enzyme that controls the ECM environment following organization of heparin sulfate in GAGs, and reports show its expression level regulates EMT [33]. A low level of *FAM198B* attenuates tumor growth and metastasis [34]. Overexpression of *IGFBP5* reduces EMT [35]. Although these reports support the notion that hub genes identified in our study actually engage in the EMT process, elucidation of the EMT mechanism remained incomplete, as clear interactions have not been defined. Given that dysregulation of ECM was found to be a profound connection with EMT [36], our EMT network indicates a possible regulatory system of interactions between ECM and EMT. Moreover, *PDK4*—identified biologically as a novel EMT-associated gene [19]—has a network location close to the hub genes, supporting our finding of including this previously validated gene and the interaction of *PDK4* with EMT-related genes.

Recently, the emergence of deep learning technology has allowed us to apply it to many scientific fields, including biology and medicine. However, such a situation depends on a large number of data sets. More importantly, the explainability of deep learning technology remains elusive. This disadvantage is a key issue, especially for biological or clinical situations, because the predictive process must also have a responsibility for interpretation and the ultimate outcome. In contrast, BN has been conventionally developed as an explainable model, and thus it is considered to be more appropriate to these fields. However, BN was unable to explain the individuality of samples and additional statistical models intended to interpret the population of certain data sets. In this study, by overcoming this weakness of BN, ECv has proven to be a more powerful tool in terms of explainability in machine learning. Therefore, the application possibilities of this BN model regarding biomedical data beyond existing bioinformatics methods makes it superior to other models. Although multi-omics analysis is becoming mainstream in biology, single transcriptome analysis still possesses a broad scope. Integration of other types of omics data, such as genome, proteome and epigenome data, with transcriptome data using BN, would lead us to analyze these big data sets more precisely than in existing studies, which in turn may result in a new approach in precision medicine.

## 5. Patents

Y.T. (Yoshinori Tamada) and Y.O. have a patent application on the individual profiling and sample specific network extraction method, and its applications used in the submitted study through the technology licensing organization in Kyoto University.

## Figures and Tables

**Table 1:** The list of concordance following different iterations.

T	Concordance
10,000	72.7%
100,000	89.0%
500,000	94.3%
1,000,000	95.6%

## Supplementary Materials

The following are available online at https://www.mdpi.com/2218-273X/10/2/306/s1: Figure S1: Venn diagram for ΔECv-extracted edges and *t*-test-significant edges. Figure S2: Venn diagram for DEG analysis. Figure S3: Unsupervised clustering and survival analysis for LUAD. Table S1: The list of the number of edges and average degrees for different thresholds. Table S2: Summary for data acquisition in this study. Table S3: The description list of supplementary files. The data in this study are available as supplementary files. The list of supplementary files is in Table S3. The ECv calculation software of the EMT network for Linux x86-64 is available to non-commercial academic users upon request.

## Abbreviations

The following abbreviations and mathematical notations are used in this manuscript:

ECv	Edge Contribution value
EMT	Epithelial-Mesenchymal Transition
BN	Bayesian Network
DEG	Differentially Expressed Gene
TCGA	The Cancer Genome Atlas project
NNSR	the Neighbor Node Sampling and Repeat algorithm
NSCLC	Non-Small Cell Lung Cancer
LUAD	Lung Adenocarcinoma
LUSC	Lung Squamous Cell Carcinoma
***X***	*n*-by-*p* data matrix whose element $x_{ij}$ corresponds to the expression value of the *j*-th gene at the *i*-th sample (samples: 1≤i≤n, genes: 1≤j≤p)
$pa_{ik}^{\left(j\right)}$	expression value of the *k*-th parent of the *j*-th gene at the *i*-th sample
θ	parameter vector of the conditional density
$m_{k}^{\left(j\right)}\left(pa_{ik}^{\left(j\right)}\right)$	value of the regression curve by *B*-splines, or contribution of the *k*-th parent of the *j*-th gene to the expression value of $x_{ij}$
$\sigma_{j}$	standard deviation for total observations of the *j*-th gene
$ECv_{\left(i\right)}\left(j_{k}\to j\right)$	edge contribution value for edge $j_{k}\to j$ with respect to the *i*-th sample
***E***	*n*-by-*m* ECv matrix whose element $e_{iv}$ corresponds to ECv of the *v*-th edge at the *i*-th sample (samples: 1≤i≤n, edges: 1≤v≤m)
