# Supplementary material for: System-Based Differential Gene Network Analysis for Characterizing a Sample-Specific Subnetwork
Source: Biomolecules. 2020 Feb 14;10(2):306. doi: 10.3390/biom10020306 (PMC7072632; doi:10.3390/biom10020306)
Supplement: Supplementary file 1 [file biomolecules-10-00306-s001.zip › Supplementary_Tanaka_proofreading/ECvpaper_supplementary_revised20200214.pdf]

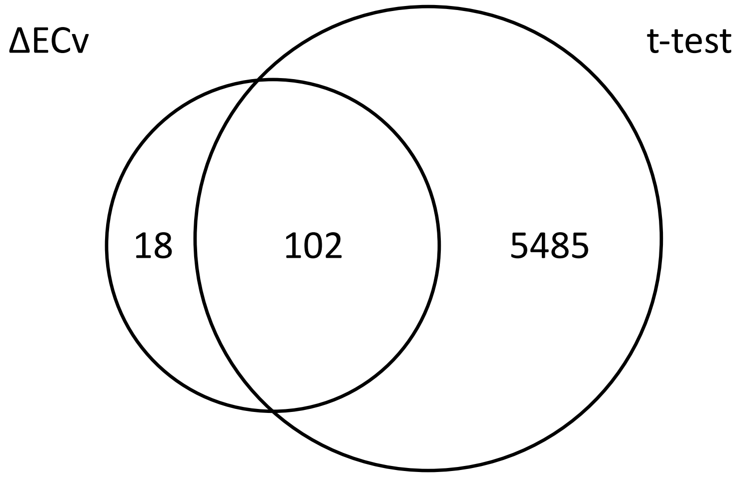

Figure S1: Venn diagram for ΔECv-extracted edges and t-test-significant edges
The t-test for ECv between TGFβ-treated (9 samples) and control (9samples) were performed. The t-test-significant edges were selected with a criteria of FDR-corrected p value < 0.01. The number of the obtained edges was displayed in Venn diagram.

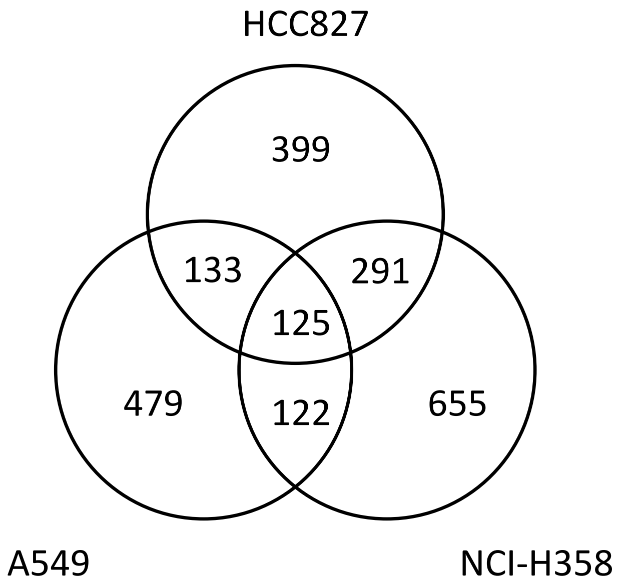

Figure S2: Venn diagram for DEG analysis
DEG analysis was performed with the criteria of absolute $\log_2 FC > 2$ and FDR-corrected p value < 0.00001 for each cell line; A549, HCC827 and NCIH-358. The number of the obtained genes was displayed in Venn diagram.

(A)

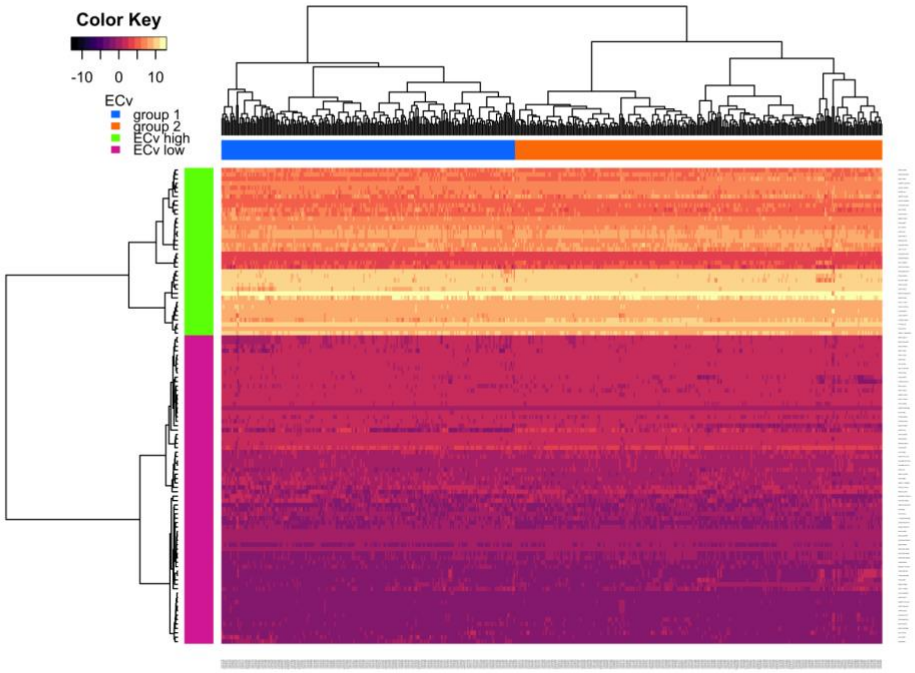

(B)

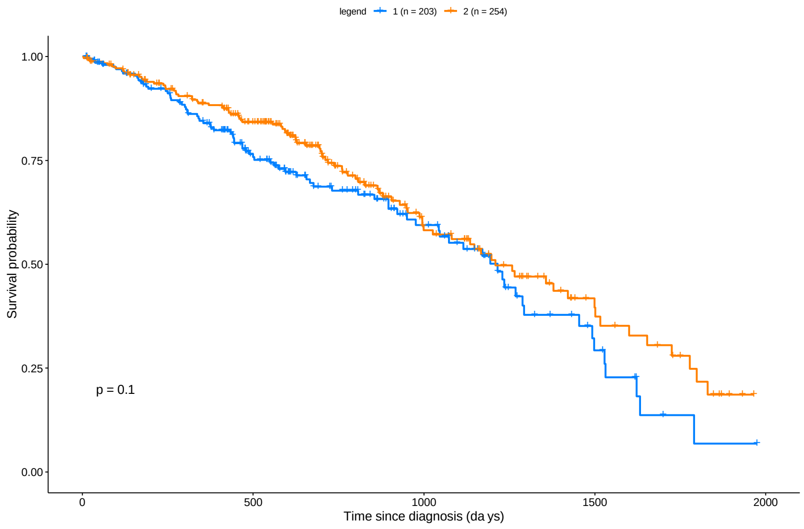

Figure S3: Unsupervised clustering and survival analysis for LUAD
(**A**) Heat map with hierarchical clustering for the ECv matrix of 108 edges with 457 samples in LUAD RNA-Seq data. (**B**) Kaplan-Meier curves for the two patient groups; group 1 (blue, n: 203) and group 2 (orange, n: 254), corresponding the patient clusters in the heat map in A. The survival analysis was performed using log rank-test for p value calculation.

Table S1: The list of the number of edges and average degrees for different thresholds

**threshold**	**edges**	**degree average**
0.05	286675	28.89
0.10	154369	15.55
0.15	101793	10.26
0.20	74085	7.46
0.30	38735	3.90

Table S2: Summary for data acquisition in this study

Data	Download source	Download data	Version	Link
Microarray GSE49644	GEO	6 Mar, 2019	-	https://www.ncbi.nlm.nih.gov/geo/query/acc.cgi?acc=GSE49644 (GSE49644_annotated_results.txt.gz)
LUSC RNA-seq	UCSC Xena	9 Dec, 2019	2017-10-13	https://xenabrowser.net/datapages/ ?dataset=TCGA.LUSC.sampleMap%2FHiSeqV2 &host=https%3A%2F%2F tcga.xenahubs.net&removeHub =https%3A%2F%2 Fxena.treehouse.gi.ucsc.edu%3A443
LUSC clinical	TCGA	9 Dec, 2019	-	https://portal.gdc.cancer.gov/projects/TCGA-LUSC
LUAD RNA-seq	UCSC Xena	9 Dec, 2019	2017-10-13	https://xenabrowser.net/datapages/ ?dataset=TCGA.LUAD.sampleMap%2FHiSeqV2 host=https%3A%2F%2F tcga.xenahubs.net&removeHub =https%3A%2F%2 Fxena.treehouse.gi.ucsc.edu%3A443
LUAD clinical	TCGA	9 Dec, 2019	-	https://portal.gdc.cancer.gov/projects/TCGA-LUAD

Table S3: The description list of supplementary files.

File	Description
GSE49644GN.txt	The basal gene network file consisting of 19,849 genes and 154,369 edges. The file is a tab separated text file. The first row is the header row representing the meanings of columns. Each line represents a single edge. The first column is a parent gene name, and the second a child gene name of an edge. The estimated *B*-spline model parameters are also included. This can be used as an input network for ECv calculation software which is provided for noncommercial, academic use upon request.
GSE49644GN_EMT.txt	The ΔECv$\geq$1.0 EMT-induced 120 edges consisting of 150 genes. The file is a tab separated text file. The first row is the header row representing the meanings of columns. Each line represents a single edge. The first column is a parent gene name, and the second a child gene name of an edge. The estimated *B*-spline model parameters are also included. This can be used as an input network for ECv calculation software mentioned above.
GSE49644_EMT_ECv.txt	ECv matrix of the EMT network with GSE49644 18 sample data. The file is tab separated text file with the header row and the label column. The header row contains the names of 18 samples. The label column contains the name (*parent-child*) of 120 edges.
basal_hubgenes_top5per.txt	The top 5% hub genes (1156) in the basal network in Figure 4. The file is tab separated text file with the header row. The header row contains the gene name and the number of outdegree.
EMT_hubgenes_top5per.txt	The top 5% hub genes (7) in the EMT network in Figure 4. The file is tab separated text file with the header row. The header row contains the gene name and the number of outdegree.
DEG_ECv_shared_genes.txt	The ΔECv and DEG shared 71 genes in Figure 5.
DEG_genes.txt	The DEG analysis extracted 125 genes in Figure 5.
ECv_matrix_TCGA_LUSC.txt	ECv matrix of 108 edges for TCGA LUSC cancer 426 patient data in Figure 6. The file is tab separated text file with the header row and the label column. The label column contains the name (*parent-child*) of 108 edges commonly exist both in the EMT network and TCGA LUSC data.
ECv_matrix_TCGA_LUAD.txt	ECv matrix of 108 edges for TCGA LUAD cancer 457 patient data in Figure S2. The file is tab separated text file with the header row and the label column. The label column contains the name (*parent-child*) of 108 edges commonly exist both in the EMT network and TCGA LUAD data.
GSE49644GN_all_EMT.cys	The basal network file for Cytoscape used in Figure 1.
GSE49644GN_EMT.cys	The EMT network file for Cytoscape used in Figure 4.
